# Reliability of Financial Information from the Perspective of Benford’s Law

**DOI:** 10.3390/e23050557

**Published:** 2021-04-30

**Authors:** Ionel Jianu, Iulia Jianu

**Affiliations:** Department of Accounting and Audit, The Bucharest University of Economic Studies, 010374 Bucharest, Romania; jianu.iulia@cig.ase.ro

**Keywords:** Benford’s Law, reliability, IFRS, financial statements, emerging capital market

## Abstract

This study investigates the conformity to Benford’s Law of the information disclosed in financial statements. Using the first digit test of Benford’s Law, the study analyses the reliability of financial information provided by listed companies on an emerging capital market before and after the implementation of International Financial Reporting Standards (IFRS). The results of the study confirm the increase of reliability on the information disclosed in the financial statements after IFRS implementation. The study contributes to the existing literature by bringing new insights into the types of financial information that do not comply with Benford’s Law such as the amounts determined by estimates or by applying professional judgment.

## 1. Introduction

The reliability of financial information is essential to ensure the credibility and stability of capital markets. The main aim of this paper is to test the reliability of financial information before and after IFRS implementation by listed companies on the Romanian capital market. Romanian capital market, which is the main entrance for Foreign Direct Investments (FDI), is becoming more and more attractive for international investors. The National Bank of Romania evaluated FDI stock on 31 December 2018 at 81.1 billion EUR, with 57.5 billion EUR in equity positions. The European Commission had estimated that the domestic economy was set to expand in 2019 by 3.3%. Meanwhile, the Eurozone was set to grow by 1.2%, while the overall growth in the EU bloc would stand at 1.4% [1]. On this backdrop, Romania will remain one of the economies with the fastest GDP expansion in the region. For this reason, this research, which tests the reliability of financial information, is very important for potential international investors.

In order to test the reliability, it is recommended to use Benford’s Law, which is an old (1938) technique that had not been used for the last 20 years. For this reason, Mir and Ausloos [2] called this technique the “Sleeping Beauty”. In the last two decades, Benford’s Law has been used more and more in the accounting field to test the reliability of financial information. Over time, accounting has undergone continuous changes in order to increase the relevance and credibility of information provided to investors. Throughout the time, accounting has suffered continuous changes in order to increase the relevance and reliability of information for investors. To what extent this desideratum has been achieved, it is the purpose of this study, by testing the information presented in financial statement by the Romanian companies listed on BSE, both before IFRS implementation (when the national accounting regulations, issued by the Romanian Finance Minister, had been applied by all companies operating in Romania) and after IFRS implementation (when the standards, issued by the International Accounting Standard Board, have become mandatory for all listed companies). The reliability testing was performed by using the first digit test of Benford’s Law, which compares the distribution of the first digit from the data set with the specific distribution of Benford’s Law. The testing was performed to the most important financial statements established by listed companies, respectively balance sheets, income statements and cash-flows statements. Conformity to Benford’s Law for the financial information means there is evidence that they do not contain errors and therefore they accurately present the reality of transactions. Because not all types of financial information are appropriate to be tested by Benford’s Law, the second aim of this paper is to identify whether the amounts based on estimates or determined using professional judgement conform or not to Benford’s Law.

Benford’s Law has its origin in the finding made by Newcomb (1881) [3], regarding the fact that the first pages of logarithmic tables contain to a greater extent numbers that have the smallest digits on the first position. Practically, independent of the finding, made by Newcomb, but based on the same argument, Benford (1938) [4] went further, testing in 20,000 observations collected from different articles, published in Reader’s Digest, the probability of occurrence of numbers that begin with each digit “1, 2, …, 9”. Since the publication of Benford’s Law, several mathematicians have presented various explanations to support Benford’s Law, but without reaching a mathematical algorithm [5].

The use of Benford’s Law has proved effective in many fields: insurance [6], databases [7,8]), economics [9,10], education [11], election [12], public administration [13], real-estate [14], religion [15], statistical studies [16,17]. In the field of finance and accounting, Benford’s Law has been applied in audits to establish specific procedures [18] and to identify potential frauds at the company level [19,20,21,22,23], as well as to combat tax evasion by identification of companies that resort to manipulating the tax base, in order to be included in a lower tax ceiling [24].

Certification of the reliability of information, disclosed in the companies’ financial statements, through the use of Benford’s Law, has been performed on capital markets of different countries: Hungary [25], India [26], Indonesia [27], Korea [28], Poland [29], Romania [30], Russia [31], Taiwan [32], Turkey [33], UK [34] and USA [35,36].

Based on the importance of financial information, in the investment-making process on the capital market, as well as in the evaluation of liquidity and solvency of companies by creditors, relevance and reliability of financial information are the fundamental qualitative characteristics [37]. If previous studies validated the hypothesis that the information presented by listed companies on Bucharest Stock Exchange (BSE) is relevant to investors [38,39], this study would confirm that this information is also reliable.

Considering the need to ensure the comparability of accounting information in the era of globalization, Romanian companies listed on the BSE have applied since 2012 the International Financial Reporting Standards (IFRS). Most studies on the relevance of financial information, performed at international level, have confirmed the superiority of the quality of information presented according to IFRS, compared to national accounting regulations. However, no study was performed to test whether the reliability of information, presented under IFRS, is superior to that available under the national accounting regulations. The results of this study confirm the increase of reliability after IFRS implementation for the items tested from balance sheets and cash flows statements. In the case of income statements, the increase of reliability after IFRS implementation was validated only in the case of companies that record profits, but not in the case of companies that record losses.

The results of this study are useful to potential investors, as well as to regulatory bodies that support the development process of the capital markets. This study can be a model for testing the reliability of information presented in financial statements of companies listed on international capital markets. The results of this study confirm the hypothesis that Benford’s Law should not be applied to items presented in the financial statements which are determined on the basis of estimates or calculated by applying the professional judgment due to the complex accounting policies.

The rest of this paper is organized as follows: Section 2 covers the literature review, Section 3 presents the research methodology, Section 4 discusses the descriptive statistics and the results, Section 5 reports the conclusions.

## 2. Literature Review

The first use of Benford’s Law in the accounting field belongs to Carslaw (1988) [40], which showed the tendency of managers to round up the profit reported by companies, if they do not reach the targets set by analysts. Carslaw’s research was conducted based on the financial data reported by New Zealand listed companies. The research conducted by Thomas (1989) [41] followed. Using Benford’s Law to test the earnings per share of US listed companies, Thomas confirmed the tendency of managers to round up earnings per share when companies recorded profits and to round down earnings per share, respectively, when companies recorded losses.

Nigrini [23,42] was the first researcher who used Benford’s Law in the process of detecting abusive result manipulation practices. Nigrini’s research [43] expanded into the field of taxation, in order to identify the companies that resorted to tax evasion practices, by developing an index that measures the percentage by which a company deviates from the correct tax base.

In a comparative analysis using the Benford’s Law, aiming to identify the effects of the Sarbanex-Osley Act on US companies, the results suggest the existence of cosmetic earnings management prior to the Act and the decrease of such behaviour after the Act [44]. The recession times affect the company activity and, for this reason, it is more difficult for management to reach the analyst’s targets, the reason for which the financial statements manipulation is larger around these time periods [45].

In order to identify the companies which are engaged in earnings management, Johnson [46] tested different characteristics using Benford’s Law, resulting in three main characteristics of these companies: capitalization below 45 billion, 3% levels of inside trading, and less than 25 years on the capital market. In addition to the previous accrual models used to identify the earnings manipulation, Lin et al. [47] found that, by using Benford’s Law, the Fat Cat companies use more earnings management than the non-Fat Cat companies, and for the latter, after they are declared to be Fat Cats, the earnings management decreases. There are also authors who show that different models, such as the Dechow model, are better than Benford’s Law in predicting the influence of earnings management to the value relevance of accounting information [48].

The use of Benford’s Law has been extended to the fraud detection process. Referring to the moment when the frauds are produced, Gava and de Souza Vitiello [21] showed that it is more probable that they will occur in the first three quarters, because they will be unnoticed compared to the situation in which they would have been recorded at the end of the reporting period. The authors also showed that the likelihood of fraud, in financial statements, is bigger in high inflation periods than in those with stable prices. Replicating and extending the Debreceny and Gray [20] study on other dataset than the US companies, Seow et al. [49] applied effectively Benford’s Law on journal entries, emphasising the usefulness of this technique for cross-sectional digital analysis of fraud. Due to the impossibility of Benford’s Law to answer the questions as “so-what”, they recommended to supplement it by other methods tested to identify the fraud. The use of Benford’s Law in fraud detection is not devoid of criticism, the most vehement being the ones regarding the Type 1 errors (eg. Cleary and Thibodeau [50]).

Durtschi et al. [22] identified the types of fraud that auditors can detect by using Benford’s Law in auditing financial statements, providing at the same time guidance on the types of data and statistical tests which auditors should use. Applying Benford’s Law in auditing the financial statements of a company from the medical industry, Asllani and Naco [51] construct a useful methodology to be used by auditors in identifying the categories of assets, liabilities, revenue and expenses which present a high risk of fraud in accounting. In their recent article, Fay and Negangard [52] present a procedure of analysis regarding the manual journal entries that could be used in the audit of financial statements to identify the potential red flags of fraud using Benford’s Law. Intensifying the use of Benford’s Law to some population of transactional data, Nigrini and Miller [53] provide a new second-order test for differences between the ordered transactions.

Most studies conducted by using Benford’s Law to test the reliability of financial information were done on the US and UK capital markets, based on accounting references, used by the listed companies in those countries, i.e., Generally Accepted Accounting Principles in the USA and IFRS in the UK. respectively. Using Benford’s Law, Archambault and Archambault [54] examined the earnings management before the pre-SEC periods in both regulated and unregulated industries, finding the presence of this practice in both types of industries, but focused on different items from the financial statements (unregulated industries managed gross revenue, net income, and accounts payable, while regulated industries managed other income, property, plant and equipment and bonds payable).

The existence of the earnings management was also confirmed by Henselmann et al. [55], the results showing that there is a high degree of deviation from Benford’s Law when the earnings beat the analyst’s targets. In contrast, other authors found a contradictory result. Therefore, Amiram et al. [35] found that financial statements of companies listed on the US capital markets conform to Benford’s Law (the first digit test was performed). Nevertheless, the amounts from income statements present larger variations from Benford’s Law than those presented in the balance sheets or cash-flows statements.

Applying Benford’s Law on US capital markets, Nigrini [36] highlights the perfect compliance of Benford’s Law in the case of stock returns, small deviations being registered only in the volume of transactions due to the rounding of amounts. The application of Benford’s Law proved to be useful in identifying variables that have the ability to predict banks failure prediction [56]. Benford’s Law was also applied to analyse the evolution of specific items presented in financial statements of the US listed companies, the results of these studies emphasizing differences regarding the conformity to Benford’s Law, between large and small companies, as well as between companies audited by Big 4 and non-Big 4 [57].

Nguyen et al. [34] tested Benford’s Law in the case of companies listed on the UK capital markets, finding the same results with Amiram et al. [35], regarding the conformity to Benford’s Law, both at the market level and within the same company, through the analysis of information over time. Furthermore, Nguyen et al. [34] identified the potential causes of the deviations registered for amounts presented in the income statements, as earnings manipulation and application of prudence principle.

## 3. Research Methodology

### 3.1. Research Model

Benford’s Law verifies the distribution of different sets of values. In order to test the reliability of information disclosed in the financial statements of companies listed on BSE, the first digit test of Benford’s Law was used. This test verifies the frequency of the digit on the first position for the data set. According to the first digit test, the percentage of the digits 1 to 9, which should appear on the first position of each number from the data set, is presented in Table 1. It is considered that as long as the analysed numbers conform to Benford’s Law, those numbers should be considered reliable for investors. The larger the data set is, the more accurate the results obtained by applying Benford’s Law are. A number of minimum 500 items is considered appropriate to apply Benford’s Law. Nevertheless, there are studies which have shown that, even in the case of applying Benford’s Law on a small number of data, of at least 50, it can be concluded that the results obtained could be correct [58]. In this study, the tested data are numerical information contained in financial statements of listed companies. Because the data set contains numbers that begin with digits 1 to 9, the basic rule to apply Benford’s Law is respected.

### 3.2. Research Hypotheses

The first objective of this study was to test the reliability of information presented in financial statements by companies listed on BSE. Since 2012, Romanian listed companies have participated in an extensive process of transition from the application of national accounting regulations to IFRS implementation. Taking into consideration the fact that IFRS are considered to be a set of high quality standards, we expect the reliability of information by IFRS implementation to be higher than that obtained by applying national accounting regulations. The hypothesis of increasing the reliability of financial information after IFRS implementation was verified using the first digit test of Benford’s Law for each of the most important financial statements established by listed companies: balance sheet, income statement and cash-flows statement. Based on the experiment of financial-accounting data and capital markets, the previous studies demonstrated that in order to apply Benford’s Law, the tested data should be extracted from a set of numbers that results from a combination of numbers [22,59]. Consequently, in order to test the reliability of information disclosed in the balance sheet, we used data calculated by the numerical combination “quantity * price”, as in the case of “accounts receivable” and “accounts payable”. Similarly, to test the reliability of information disclosed in the income statement, we used performance indicators, calculated by the numerical combination “revenue expenses”, as in the case of “net income”. In the end, to test the reliability of information disclosed in the cash-flows statement, we used the performance indicators calculated by the numerical combination “receipts payments”, as in the case of “cash from operating activities”. Therefore, to test the reliability of information disclosed in each of the major financial statements by using Benford’s Law, three hypotheses, which we expect to validate, were formulated:

**Hypothesis** **1.** *The reliability of information disclosed in the balance sheet increased after IFRS implementation*.

**Hypothesis** **2.** *The reliability of information disclosed in the income statement increased after IFRS implementation.*

**Hypothesis** **3.** *The reliability of information disclosed in the cash-flows statement increased after IFRS implementation.*

The second objective of this study was to confirm that Benford’s Law should not be applied to test the items in financial statements, whose values are determined by estimates. According to Nigrini and Mittermaier [18], Benford’s Law is not recommended to be applied when human thought is involved. The financial statements items, most likely to be influenced by the accountant’s professional judgment, are the depreciation expenses. This happens because their calculation depends on many subjective aspects related to: estimating the economic useful life of fixed assets, choosing the depreciation method (which is influenced by the consumption of future economic benefits), and establishing the residual value by estimating future gains generated by value recovery at the end of use. Consequently, to confirm the rule that Benford’s Law should not be applied to test items in financial statements, whose values are influenced by human thoughts, the following hypothesis, which we expect to validate, was formulated:

**Hypothesis** **4.** *Distributions of the first digits of depreciation expenses reported in the income statement do not follow Benford’s Law.*

Testing this hypothesis will be based on data regarding the depreciation expenses presented in the income statements of Romanian companies listed on BSE. The data, used to test Benford’s Law, were calculated both in accordance with national accounting regulations and IFRS.

The third objective of this study was to demonstrate that Benford’s Law should not be applied to test the items assessed and recognised using complex accounting standards that involve the use of extensive accountant’s professional judgment, which leads to different practices of applying accounting regulations. An example, in this case, will occur if the standard IFRS 15 “Revenue from contracts with customers” is applied. This is a very complex standard regarding revenue recognition which involves the extensive use of the accountant’s professional judgment. In order to verify this hypothesis, it was selected the item “Revenue” from the income statement. Consequently, to demonstrate the fact that Benford’s Law should not be applied to test items in financial statements, whose values are assessed and recognised by using extensively the accountant’s professional judgment, the following hypothesis, which we expect to validate, was formulated:

**Hypothesis** **5.** *Distributions of the first digits of revenue reported in the income statement after IFRS implementation do not follow Benford’s Law.*

We expect this hypothesis to be validated in the case of IFRS implementation and invalidated when national accounting regulations were applied, because, in this last case, there are very clear rules for revenue recognition. To strengthen the belief that Benford’s Law should not be applied to test amounts calculated by using very complex accounting treatments, but only the amounts calculated using clearly defined rules, the income tax expenses were extracted from the data set in order to be analysed. This item was selected because the income tax expenses were calculated by applying the rules which are very clearly defined in the Tax Code. In the case of income tax expenses, we expect the distributions of first digits to follow Benford’s Law both before and after IFRS implementation.

### 3.3. Data Collection

In order to test the hypotheses, data published by Romanian companies listed on BSE were used. The data were extracted from Thomson Reuters Eiken database. At the time of data extraction (February 2019), 85 companies were listed on BSE. For a number of seven companies, the data was unavailable, three companies presented data in a currency other than the Romanian currency (RON), and three companies are banks (financial statements for these companies have a different structure due to the specifics of their activity); therefore, these 13 companies were eliminated from the study. Consequently, data from a number of 72 companies were analysed. These companies present data from financial statements starting with 1996 and until 2017. The companies listed on BSE after the year 1996 present data starting with the year in which they were listed on BSE.

Because one of the research objectives was to test the reliability of information disclosed in income statements after IFRS implementation, data collected for each analysed item was separated in two categories: items calculated and presented by companies before IFRS implementation (when the national accounting regulations were applied) and after IFRS implementation. For the majority of companies, the effective implementation of IFRS was realized in 2012. For the year prior to effective application, in order to provide comparative information, companies established financial statements, both according to national accounting regulations and IFRS. Within the analysed companies, a number of 15 companies made the transition to IFRS earlier than 2012.

Nigrini [43] considers that data whose value is lower than 50 should be eliminated from the analysis because they are immaterial and might introduce bias in the results. Taking into consideration that data extracted from Thomson Reuters Eiken database are expressed in thousands RON, when the first digit test was applied, in order to avoid the cases when the first digit was obtained by rounding, the data of which amount is less than 10 were deleted.

Since the data used to test the conformity to Benford’s law for all digits are categorical data, the chi-square test was used to support the results, similarly with other studies [20,54,60,61]. When the percentage distribution of data set is closer to the standard Benford’s Law distribution, it is assumed that the reliability of financial information is bigger. To explore the robustness of the results, the other tests were used to verify the conformity to Benford’s Law, as the mean absolute deviation (MAD) and the Kolmogorov-Smirnov (KS) test. The MAD does not depend on the sample size. For this reason, it is recommended to be used for testing large data sets (more than dozens of thousands of data, which is not the case of the present research). The KS test becomes more sensitive as data series grow. Therefore, the KS test is most appropriate to test the conformity to Benford’s Law for small data sets. Given that the number of observations used in this research to test compliance to Benford’s Law varies between 93 and 686 observations, Chi-square was chosen to test the first digits for all observations, at a confidence level of 95%. In order to test the conformity to each digit, Z test was used, similarly with other studies [28,40,62,63].

## 4. Results

**Figure 1:**
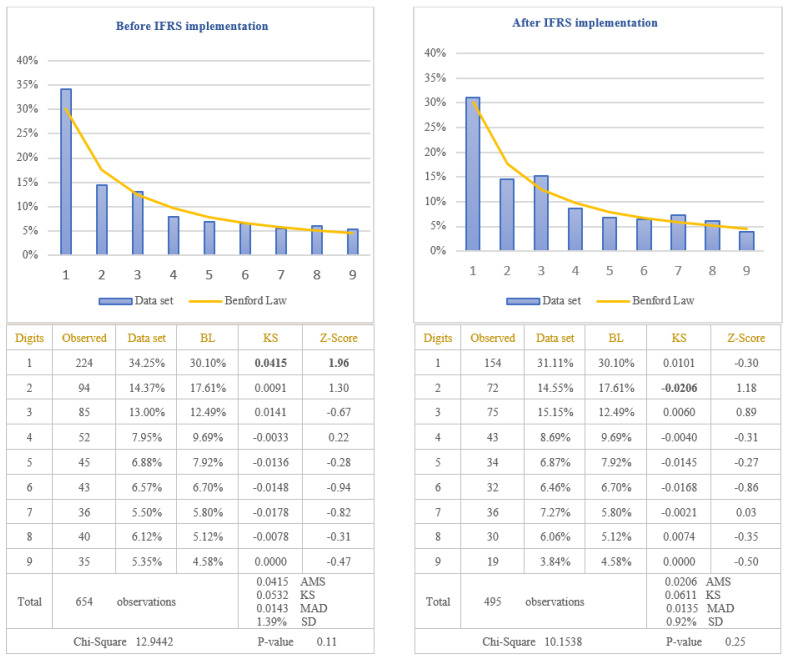
Accounts receivable. The figure shows the conformity to Benford’s Law of Accounts Receivable in accordance with Chi-square test, both before and after IFRS implementation. Score based on Chi-Square of 12.9442 (*p*-value 0.11) before IFRS implementation and 10.1538 (*p*-value 0.25) after IFRS implementation is lower than the critical value of 15.5073 at 5% level. Data set columns represents the observed percentages of the first digits and BL columns represents Benford’s Law expected percentages of the first digits. Before IFRS implementation, the absolute maximum score (AMS) of 0.0415 based on Kolmogorov-Smirnov is lower than the test value of 0.0532 at 5% level for 654 observations; after IFRS implementation, the AMS of 0.0206, based on Kolmogorov-Smirnov, is lower than the test value of 0.0611 at 5% level for 495 observations; this shows the conformity to Benford’s Law of Accounts Receivable in accordance with Kolmogorov-Smirnov test, both before and after IFRS implementation. Score based on the mean absolute deviation (MAD) of 0.0143 before IFRS implementation and 0.0135 after IFRS implementation is lower than 0.0150 (MAD bigger than 0.0150 shows the nonconformity to Benford’s Law in accordance with Nigrini [64]). Standard deviation of differences between data set and Benford’s Law distributions is 1.39% before IFRS implementation and 0.92% after IFRS implementation. Z-Score is calculated for each digit, similarly with the studies of Lacina et al. [28], Carslaw [40], Costa et al. [62] and Santos et al. [63].

**Figure 2:**
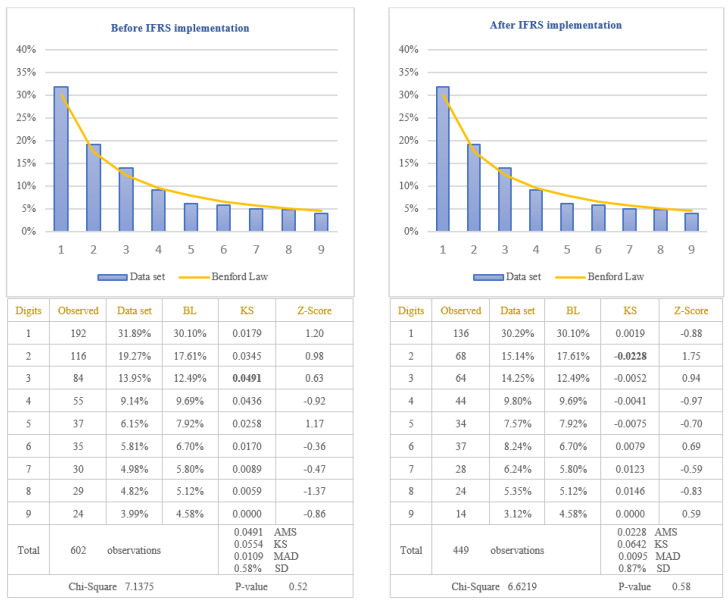
Accounts payable. The figure shows the conformity to Benford’s Law of Accounts Payable in accordance with Chi-square test, both before and after IFRS implementation. Score based on Chi-Square of 7.1375 (*p*-value 0.52) before IFRS implementation and 6.6219 (*p*-value 0.58) after IFRS implementation is lower than the critical value of 15.5073 at 5% level. Data set columns represents the observed percentages of the first digits and BL columns represents Benford’s Law expected percentages of the first digits. Before IFRS implementation, the absolute maximum score (AMS) of 0.0491 based on Kolmogorov-Smirnov is lower than the test value of 0.0554 at 5% level for 602 observations; after IFRS implementation, the AMS of 0.0228, based on Kolmogorov-Smirnov, is lower than the test value of 0.0642 at 5% level for 449 observations this shows the conformity to Benford’s Law of Accounts Payable in accordance with Kolmogorov-Smirnov test, both before and after IFRS implementation. Score based on the mean absolute deviation (MAD) of 0.0109 before IFRS implementation and 0.0095 after IFRS implementation is lower than 0.0150 (MAD bigger than 0.0150 shows the nonconformity to Benford’s Law in accordance with Nigrini [64]). Standard deviation of differences between data set and Benford’s Law distributions is 1.58% before IFRS implementation and 0.87% after IFRS implementation. Z-Score is calculated for each digit, similarly with the studies of Lacina et al. [28], Carslaw [40], Costa et al. [62] and Santos et al. [63].

The analysis of the reliability of information disclosed in the balance sheets of the companies listed on BSE was performed by applying the first digit test of Benford’s Law to the amounts of “Accounts receivable”, presented in the assets category, and to the amounts of “Accounts payable”, presented in the liability category. Both items respected the rule imposed by Benford’s Law, which involves to test the numbers that result from a numerical combination: “quantity sold * selling price” in the case of accounts receivable, respectively, “quantity purchased * purchasing price” in the case of accounts payable. Because the IFRS are considered a set of high quality standards at the international level, in order to verify if IFRS implementation increased the reliability of information disclosed in the balance sheets, data would be divided in two categories: before IFRS implementation and after IFRS implementation. The results obtained for the balance sheet items, tested by Benford’s Law, are presented in Figure 1 for the item “Accounts receivable” and in Figure 2 for the item “Accounts payable”. The figures present the results both before IFRS implementation and after IFRS implementation.

From the analysis of the first digit test, it is observed that the items “Accounts receivable” and “Accounts payable” conform to Benford’s Law both before and after IFRS implementation, at 5% level (critical value of Chi-Square 15.5073). The first digit of data, after IFRS implementation, conforms to Benford’s Law for accounts receivable (Chi-Square 10.1538) and accounts payable (Chi-Square 6.6219), more closely than the first digit before IFRS implementation for accounts receivable (Chi-Square 12.9442) and accounts payable (Chi-Square 7.1375). This suggests that assets and liabilities disclosed in the balance sheets of the companies listed on BSE are more reliable after IFRS implementation than before IFRS implementation. Analysing Z-score for each specific first digit, there is no significant difference from Benford’s Law for any of the first digit of the accounts receivable and accounts payable amounts, at 5% level. Taking into consideration the results obtained from the analysis of accounts receivable and accounts payable, we validate the Hypothesis 1 that the reliability of information disclosed in the balance sheet increased after IFRS implementation.

**Figure 3:**
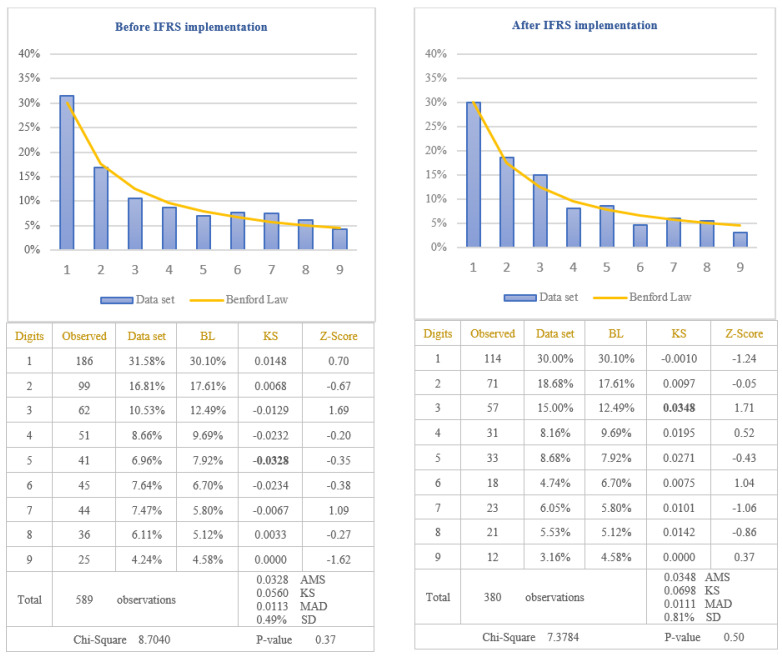
Positive amounts of Net income (Profit). This figure shows the conformity to Benford’s Law of positive amounts of Net Income in accordance with Chi-square test, both before and after IFRS implementation. Score based on Chi-Square of 8.7040 (*p*-value 0.37) before IFRS implementation and 7.3784 (*p*-value 0.50) after IFRS implementation is lower than the critical value of 15.5073 at 5% level. Data set columns represents the observed percentages of the first digits and BL columns represents Benford’s Law expected percentages of the first digits. Before IFRS implementation, the absolute maximum score (AMS) of 0.0328 based on Kolmogorov-Smirnov is lower than the test value of 0.0560 at 5% level for 589 observations; after IFRS implementation, the AMS of 0.0348, based on Kolmogorov-Smirnov, is lower than the test value of 0.0698 at 5% level for 380 observations; this shows the conformity to Benford’s Law of positive amounts of Net Income in accordance with Kolmogorov-Smirnov test, both before and after IFRS implementation. Score based on the Mean Absolute Deviation (MAD) of 0.0113 before IFRS implementation and 0.0111 after IFRS implementation is lower than 0.0150 (MAD bigger than 0.0150 shows the nonconformity to Benford’s Law in accordance with Nigrini [64]). Standard deviation of differences between data set and Benford’s Law distributions is 0.49% before IFRS implementation and 0.81% after IFRS implementation. Z-Score is calculated for each digit, similarly with the studies of Lacina et al. [28], Carslaw [40], Costa et al. [62] and Santos et al. [63].

**Figure 4:**
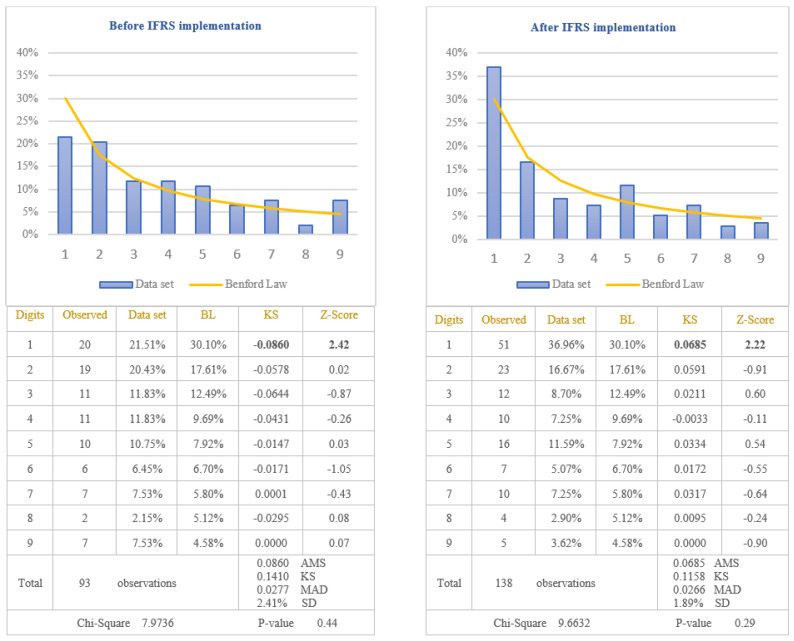
Negative amounts of Net income (Loss). The figure shows the conformity to Benford’s Law of negative amounts of Net Income in accordance with Chi-square test, both before and after IFRS implementation. Score based on Chi-Square of 7.9736 (*p*-value 0.44) before IFRS implementation and 9.6632 (*p*-value 0.29) after IFRS implementation is lower than the critical value of 15.5073 at 5% level. Data set columns represents the observed percentages of the first digits and BL columns represents Benford’s Law expected percentages of the first digits. Before IFRS implementation, the absolute maximum score (AMS) of 0.0860 based on Kolmogorov-Smirnov is lower than the test value of 0.1410 at 5% level for 93 observations; after IFRS implementation, the AMS of 0.0685, based on Kolmogorov-Smirnov, is lower than the test value of 0.1158 at 5% level for 138 observations; this shows the conformity to Benford’s Law of negative amounts of Net Income in accordance with Kolmogorov-Smirnov test, both before and after IFRS implementation. Score based on the Mean Absolute Deviation (MAD) of 0.0277 before IFRS implementation and 0.0266 after IFRS implementation is bigger than 0.0150 (MAD bigger than 0.0150 shows the nonconformity to Benford’s Law in accordance with Nigrini [64]). Standard deviation of differences between data set and Benford’s Law distributions is 2.41% before IFRS implementation and 1.89% after IFRS implementation. Z-Score is calculated for each digit, similarly with the studies of Lacina et al. [28], Carslaw [40], Costa et al. [62] and Santos et al. [63].

The analysis of the reliability of information disclosed in the income statements of the companies listed on BSE was performed by applying the first digit test of Benford’s Law to the amounts of “Net income”, calculated by difference between total revenues and total expenses, recorded during the financial year. Taking into consideration the fact that net income can be positive when revenues are bigger than expenses (in this case the company obtained profit) or negative when revenues are lower than expenses (in this case the company obtained a loss), the data set was divided in two categories: positive amounts versus negative amounts. This separation is imposed when Benford’s Law test is used because the incentive to manipulate the earnings will be different if the company obtains profit or loss. Therefore, if the company has obtained profit, the incentive would be to increase the amount of profit, but if the company has obtained a loss, the incentive would be to decrease the amount of loss, in order to be very close to zero. Because the objective of this study is to test the reliability of financial information after IFRS implementation, the data which reflect the amounts of profit, respectively the amounts of loss, were also divided in two categories: before and after IFRS implementation. The results obtained by testing the data are presented in Figure 3 for the amounts expressing the profit before and after IFRS implementation, and in Figure 4 for the amounts expressing the loss before and after IFRS implementation.

From the analysis of the first digit test, it is observed that the item “Net income” conforms to Benford’s Law both before and after IFRS implementation, at 5% level (critical value of Chi-Square 15.5073). The first digit of profit amounts after IFRS implementation conforms to Benford’s Law (Chi-Square 7.3784) more closely than the first digit before IFRS implementation (Chi-Square 8.7040). On the contrary, the first digit of loss before IFRS implementation (Chi-Square 7.9736) conforms to Benford’s Law more closely than the first digit after IFRS implementation (Chi-Square 9.6632). This indicates that the amounts disclosed in the income statements of the companies listed on BSE are more reliable after IFRS implementation than before IFRS implementation, when companies obtained profit, but not in the case when companies obtained losses. Analyzing Z-scores for each specific first digit, it is highlighted the fact that there are significant differences from Benford’s Law for digit 1 when the companies obtained losses, both before and after IFRS implementation, at 5% level (critical value of Z-score 1.96). The main reason for these differences is the small number of observations available in the case of negative amounts for net income (93 observations before IFRS implementation and 138 observations after IFRS implementation). Taking into consideration the results obtained from the analysis of the amounts of net income, we validate the Hypothesis 2 that the reliability of information disclosed in the income statement increased after IFRS implementation, only for positive amounts of net income, but not for negative amounts of net income. We could conclude that the reliability of information disclosed in income statements increased after IFRS implementation only for the profitable companies.

**Figure 5:**
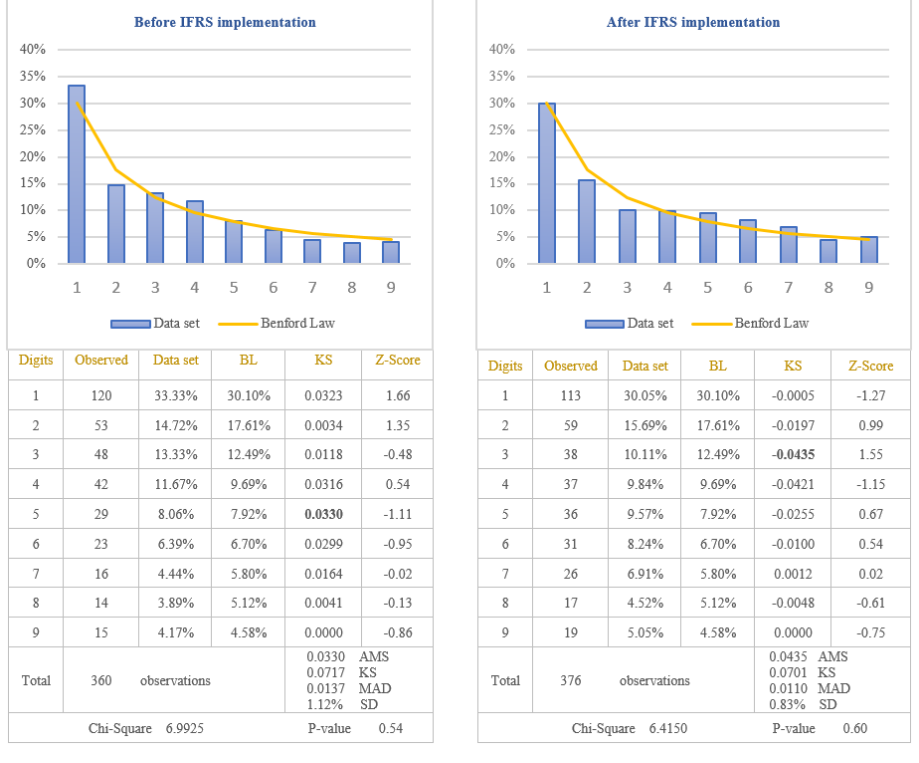
Positive cash-flows from operating activities. The figure shows the conformity to Benford’s Law of positive Cash-flows from operating activities in accordance with Chi-square test, both before and after IFRS implementation. Score based on Chi-Square of 6.9925 (*p*-value 0.54) before IFRS implementation and 6.4150 (*p*-value 0.60) after IFRS implementation is lower than the critical value of 15.5073 at 5% level. Data set columns represents the observed percentages of the first digits and BL columns represents Benford’s Law expected percentages of the first digits. Before IFRS implementation, the absolute maximum score (AMS) of 0.0330 based on Kolmogorov-Smirnov is lower than the test value of 0.0717 at 5% level for 360 observations; after IFRS implementation, the AMS of 0.0435, based on Kolmogorov-Smirnov, is lower than the test value of 0.0701 at 5% level for 376 observations; this shows the conformity to Benford’s Law of positive Cash-flows from operating activities in accordance with Kolmogorov-Smirnov test, both before and after IFRS implementation. Score based on the mean absolute deviation (MAD) of 0.0137 before IFRS implementation and 0.0110 after IFRS implementation is lower than 0.0150 (MAD bigger than 0.0150 shows the nonconformity to Benford’s Law in accordance with Nigrini [64]). Standard deviation of differences between data set and Benford’s Law distributions is 1.12% before IFRS implementation and 0.83% after IFRS implementation. Z-Score is calculated for each digit, similarly with the studies of Lacina et al. [28], Carslaw [40], Costa et al. [62] and Santos et al. [63].

**Figure 6:**
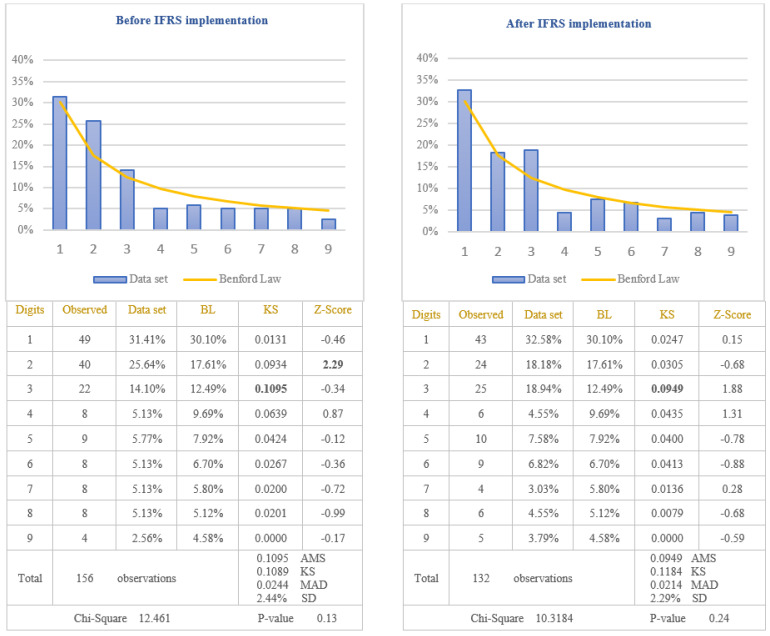
Negative cash-flows from operating activities. The figure shows the conformity to Benford’s Law of negative Cash-flows from operating activities in accordance with Chi-square test, both before and after IFRS implementation. Score based on Chi-Square of 12.461 (*p*-value 0.13) before IFRS implementation and 10.3184 (*p*-value 0.24) after IFRS implementation is lower than the critical value of 15.5073 at 5% level. Data set columns represents the observed percentages of the first digits and BL columns represents Benford’s Law expected percentages of the first digits. Before IFRS implementation, the absolute maximum score (AMS) of 0.1095 based on Kolmogorov-Smirnov is bigger than the test value of 0.1089 at 5% level for 156 observations; after IFRS implementation, the AMS of 0.0949, based on Kolmogorov-Smirnov, is lower than the test value of 0.1184 at 5% level for 132 observations; this shows the conformity to Benford’s Law of negative Cash-flows from operating activities in accordance with Kolmogorov-Smirnov test only after IFRS implementation. Score based on the mean absolute deviation (MAD) of 0.0244 before IFRS implementation and 0.0214 after IFRS implementation is bigger than 0.0150 (MAD bigger than 0.0150 shows the nonconformity to Benford’s Law in accordance with Nigrini [64]). Standard deviation of differences between data set and Benford’s Law distributions is 2.44% before IFRS implementation and 2.29% after IFRS implementation. Z-Score is calculated for each digit, similarly with the studies of Lacina et al. [28], Carslaw [40], Costa et al. [62] and Santos et al. [63].

The analysis of the reliability of information disclosed in the cash-flows statements of the companies listed on BSE was performed by applying the first digit test of Benford’s Law to the item named “Cash from operating activities” which is one of the most important indicators used by investors to evaluate the liquidity of the company. Taking into consideration that cash-flows from operating activities are calculated as a difference between receipts and payments, there is the possibility for this indicator to be negative when payments are bigger than receipts. Because previous studies have shown that the tendency to manipulate accounting data is different in the case of negative values compared to positive ones, in order to verify if the reliability of information disclosed in the cash-flows statements increased after IFRS implementation, the results are presented in Figure 5 for positive amounts of cash-flows and in Figure 6 for negative amounts of cash-flows.

From the analysis of the first digit test, it is observed that the item “Cash from operating activities” conforms to Benford’s Law both before and after IFRS implementation, at 5% level (critical value of Chi-Square 15.5073). The first digit of positive and negative amounts of cash-flows from operations after IFRS implementation (Chi-Square 6.4150 for positive amounts and Chi-Square 10.3184 for negative amounts) conforms to Benford’s Law more closely than the first digit before IFRS implementation (Chi-Square 6.9925 for positive amounts and Chi-square 12.461 for negative amounts). This indicates that positive and negative amounts of cash-flows from operations, disclosed in the cash-flows statements of the companies listed on BSE, are more reliable after IFRS implementation than before IFRS implementation. Analysing Z-score for each specific first digit, it is highlighted the fact that there are significant differences from Benford’s Law for digit 2 of the negative amounts of cash-flows before IFRS implementation, at 5% level (critical value of Z-score 1.96). The main reason for this difference is the small number of data available for testing negative amounts of cash-flows from operations (156 observations before IFRS implementation and 132 observations after IFRS implementation). Taking into consideration the results obtained from the analysis of positive and negative amounts of cash-flows from operations, we validate the Hypothesis 3 that the reliability of information disclosed in the cash-flows statement increased after IFRS implementation.

**Figure 7:**
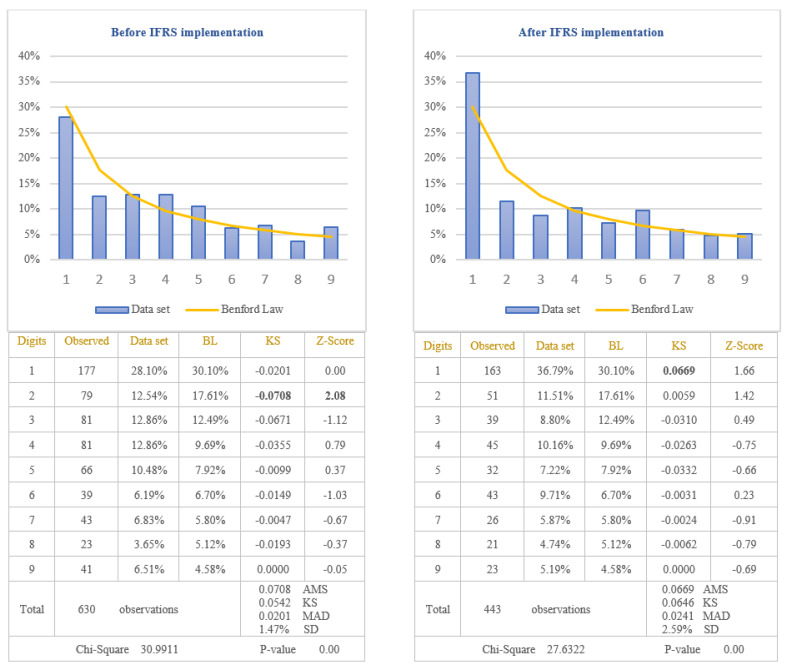
Depreciation expenses. The figure shows the nonconformity to Benford’s Law of Depreciation Expenses in accordance with Chi-square test, both before and after IFRS implementation. Score based on Chi-Square of 30.9911 (*p*-value 0.00) before IFRS implementation and 27.6322 (*p*-value 0.00) after IFRS implementation is bigger than the critical value of 15.5073 at 5% level. Data set columns represents the observed percentages of the first digits and BL columns represents Benford’s Law expected percentages of the first digits. Before IFRS implementation, the absolute maximum score (AMS) of 0.0708 based on Kolmogorov-Smirnov is bigger than the test value of 0.0542 at 5% level for 630 observations; after IFRS implementation, the AMS of 0.0669, based on Kolmogorov-Smirnov, is bigger than the test value of 0.0646 at 5% level for 443 observations; this shows the nonconformity to Benford’s Law of Depreciation Expenses in accordance with Kolmogorov-Smirnov test, both before and after IFRS implementation. Score based on the mean absolute deviation (MAD) of 0.0201 before IFRS implementation and 0.0241 after IFRS implementation is bigger than 0.0150 (MAD bigger than 0.0150 shows the nonconformity to Benford’s Law in accordance with Nigrini [64]). Standard deviation of differences between data set and Benford’s Law distributions is 1.47% before IFRS implementation and 2.59% after IFRS implementation. Z-Score is calculated for each digit, similarly with the studies of Lacina et al. [28], Carslaw [40], Costa et al. [62] and Santos et al. [63].

The second objective of this study was to demonstrate that Benford’s Law should not be applied in testing the values calculated based on estimates. The item selected to test the nonconformity to Benford’s Law for these types of values was “Depreciation expenses”. This item was selected due to the large number of estimates made to determine the value of depreciation, among which we mention: estimating the economic useful life to determine the depreciation period of time, estimating how future economic benefits will be consumed to determine the depreciation method, estimating the residual value of the asset at the end of useful life to determine the depreciable amount, estimating the recoverable value if the asset is impaired, estimating the fair value if the asset is revalued. The depreciation expenses, extracted from the income statements of the companies listed on BSE, were divided in two categories: before and after IFRS implementation. The results obtained by applying the first digit test are presented in Figure 7.

From the analysis of differences between the distribution of the first digit of the item “Depreciation Expenses” from all set of data and the standard distribution of Benford’s Law results that the differences are significant at 5% level (critical value of Chi-Square 15.5073), both before and after IFRS implementation. The first digit of data before IFRS implementation does not conform to Benford’s Law (Chi-Square 30.9911), even if only for a single digit, respectively digit 2, the conformity to Benford’s Law seems not to be respected. Regarding the data after IFRS implementation, the first digit for all data does not conform to Benford’s Law (Chi-Square 27.6322), even if each digit analysed individually conforms to Benford’s Law at 5% level. Taking into consideration the results obtained, we validate the Hypothesis 4 that the distributions of the first digits of depreciation expenses reported in income statements do not follow Benford’s Law. We could conclude that the values reported in the financial statements based on estimates do not seem to conform to Benford’s Law.

**Figure 8:**
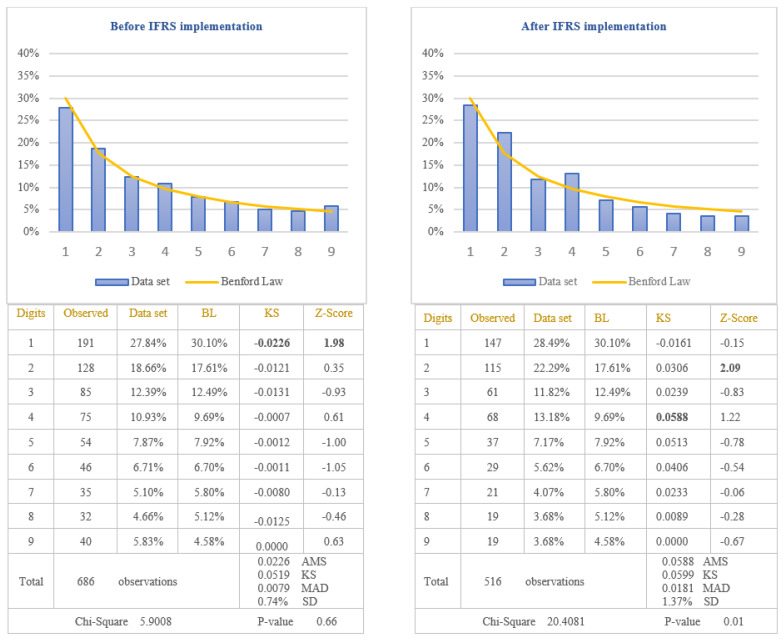
Revenues. The figure shows, in accordance with Chi-square test, the conformity to Benford’s Law of Revenues before IFRS implementation and the nonconformity to Benford’s Law of Revenues after IFRS implementation. Score based on Chi-Square of 5.9008 (*p*-value 0.66) before IFRS implementation is lower than the critical value of 15.5073 at 5% level and score based on Chi-Square of 20.4081 (*p*-value 0.01) after IFRS implementation is bigger than the critical value of 15.5073 at 5% level. Data set columns represents the observed percentages of the first digits and BL columns represents Benford’s Law expected percentages of the first digits. Before IFRS implementation, the absolute maximum score (AMS) of 0.0226 based on Kolmogorov-Smirnov is lower than the test value of 0.0519 at 5% level for 686 observations; after IFRS implementation, the AMS of 0.0588, based on Kolmogorov-Smirnov, is lower than the test value of 0.0599 at 5% level for 516 observations; this shows the conformity to Benford’s Law of Revenues in accordance with Kolmogorov-Smirnov test, both before and after IFRS implementation. Score based on the mean absolute deviation (MAD) of 0.0079 before IFRS implementation is lower than 0.015 and score based on the MAD of 0.0181 after IFRS implementation is bigger than 0.0150 (MAD bigger than 0.0150 shows the nonconformity to Benford’s Law in accordance with Nigrini [64]). Standard deviation of differences between data set and Benford’s Law distributions is 0.74% before IFRS implementation and 1.37% after IFRS implementation. Z-Score is calculated for each digit, similarly with the studies of Lacina et al. [28], Carslaw [40], Costa et al. [62] and Santos et al. [63].

**Figure 9:**
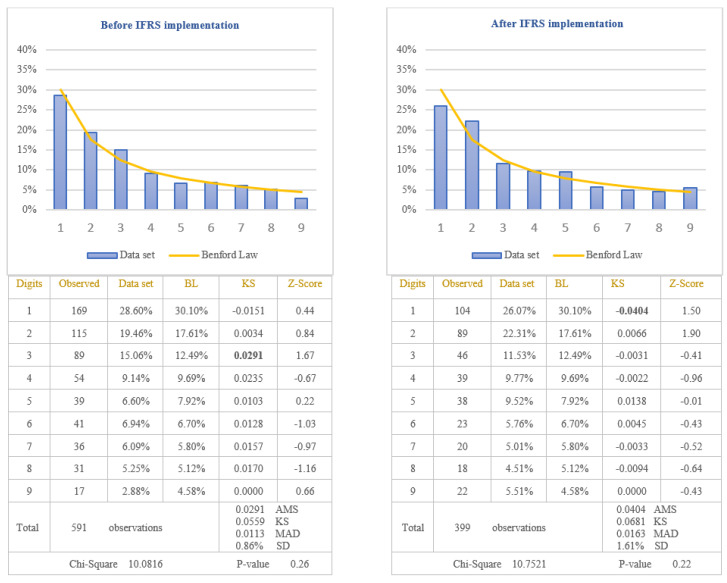
Income tax expenses. The figure shows the conformity to Benford’s Law of Income Tax Expenses in accordance with Chi-square test, both before and after IFRS implementation. Score based on Chi-Square of 10.0816 (*p*-value 0.26) before IFRS implementation and 10.7521 (*p*-value 0.22) after IFRS implementation is lower than the critical value of 15.5073 at 5% level. Data set columns represents the observed percentages of the first digits and BL columns represents Benford’s Law expected percentages of the first digits. Before IFRS implementation, the absolute maximum score (AMS) of 0.0291 based on Kolmogorov-Smirnov is lower than the test value of 0.0559 at 5% level for 591 observations; after IFRS implementation, the AMS of 0.0404, based on Kolmogorov-Smirnov, is lower than the test value of 0.0681 at 5% level for 399 observations; this shows the conformity to Benford’s Law of Income Tax Expenses in accordance with Kolmogorov-Smirnov test, both before and after IFRS implementation. Score based on the mean absolute deviation (MAD) of 0.0113 before IFRS implementation and 0.0163 after IFRS implementation is lower than 0.0150 (MAD bigger than 0.0150 shows the nonconformity to Benford’s Law in accordance with Nigrini [64]). Standard deviation of differences between data set and Benford’s Law distributions is 0.86% before IFRS implementation and 1.61% after IFRS implementation. Z-Score is calculated for each digit, similarly with the studies of Lacina et al. [28], Carslaw [40], Costa et al. [62] and Santos et al. [63].

The third objective of this study was to test if the extensive professional judgement used in IFRS impacts on the application of Benford’s Law. IFRS 15, which is the standard for revenue recognition, is considered to have a high degree of complexity [65]. Its implementation requires the use of professional judgment by accountants, which can lead to various revenue recognition practices for companies applying IFRS. These practices can affect the revenue amounts recognized in the income statement, leading to non-compliance with Benford’s Law. The testing of this hypothesis was performed by analysing the way in which the revenues recognized in the income statements of companies listed on BSE comply with Benford’s Law after IFRS implementation. To strengthen the way in which the extensive professional judgement used in IFRS affects the application of Benford’s Law, we also applied the test for the income tax expenses. In the case of income tax expenses, there are very strict rules for calculating the amount of income tax, and therefore, we expect that these amounts to conform to Benford’s Law. The results obtained by testing the conformity to Benford’s Law are presented in Figure 8 for revenues and in Figure 9 for income tax expenses.

Before IFRS implementation, from the analysis of the first digit test, it is noticed that the item “Revenue” conforms to Benford’s Law at 5% level (Chi-Square 5.9008) due to the clear rules regarding revenue recognition defined in the national accounting regulation used by Romanian companies listed on BSE. Therefore, applying the first digit test to analyse the compliance with Benford’s Law of the item “Revenue” recognized in income statement after IFRS implementation, it is noticed the non-conformity to Benford’s Law at 5% level (Chi-Square 20.4081). The digit that generates significant differences is the digit 2 for which the Z-statistic is bigger than the critical value of 1.96 at 5% level. By applying the first digit test in the case of income tax expenses, it is observed that at 5% level, the amounts of income tax conform to Benford’s Law, both before IFRS implementation (Chi-Square 10.0816) and after IFRS implementation (Chi-Square 10.7521). Taking into consideration the results obtained, we validate the Hypothesis 5 that the distributions of the first digits of revenue reported in the income statement after IFRS implementation do not follow Benford’s Law and invalidate the same hypothesis for the revenue recognised before IFRS implementation. We could conclude that the excessive use of professional judgement due to the complexity of IFRS leads to the non-compliance with Benford’s Law.

The results obtained by applying the MAD and KS test are similar to those obtained by applying the Chi-square test, with the exception of the negative cash-flows by applying MAD, the negative amount of cash-flows before IFRS implementation by applying KS, and the revenue after IFRS implementation by applying KS. Taking into consideration the superiority of the Chi-square test, in comparison with the MAD and KS for the present study, the explanations of the results are based on the Chi-square test.

## 5. Conclusions

Reliability is one of the most important qualitative characteristics of financial information. Over time, accounting has undergone a long process of transformation in order to provide high quality standards. IFRS are used by listed companies all over the word to prepare financial statements. The transition from national accounting regulations to IFRS has been made in order to provide comparative information, which is more reliable and more relevant. One of the main objectives of this study was to analyse if the transition to IFRS provided more reliable information than that obtained by applying national accounting regulations. In order to reach this objective, Benford’s Law was used to test the reliability of information, disclosed in financial statements by the companies listed on BSE. The most significant items disclosed in the financial statements prepared both before and after IFRS implementation were tested. The results of this study confirm the increase of reliability of information disclosed in the balance sheets and the cash-flows statements of listed companies after IFRS implementation. Regarding the increase of reliability of information disclosed in the income statements of listed companies, the results of this study confirm the increase in reliability after IFRS implementation only in the case of profitable companies, but not for the companies that recorded losses. This study can be used as a model for testing the reliability of information presented in financial statements on various capital markets.

Another objective of this study was to identify the type of information, disclosed in financial statements, that are suitable to conform with Benford’s Law. The results of this study highlighted the fact that Benford’s Law should not be applied in testing the amounts determined by estimates or by using the professional judgement of the accountant, as in the case of depreciation or revenue recognition. When the rules applied to calculate the amounts disclosed in financial statements are very clearly defined, those amounts tend to conform to Benford’s Law. These results strengthen the belief that subjectivity introduced in assessment, recognition and presentation of assets, liabilities, revenue and expenses in financial statements leads to data that do not seem to conform to Benford’s Law. It is known that Benford’s Law is often used as an analytical tool in the audit process to test the potential fraudulent financial statements. The conclusions of this study are very important because they reveal that non-compliance with Benford’s Law is not necessarily the result of fraud, but the fact that only certain types of data are adapted to be tested according to Benford’s Law.

As limitations of this research we can mention the small number of observations used due to the small number of companies that are listed on BSE which is considered an emerging market. It is recommended to conduct a similar research on developed capital markets to test the extent to which the maturity of the capital market can influence the reliability of accounting information. For the future researches in this field, we recommend testing Benford’s Law taking into consideration different characteristics of listed companies such as industry, size, performance indicators in order to identify whether or not they can influence Benford’s Law compliance.

## Figures and Tables

**Table 1:** Benford’s Law: Expected Digits Frequencies.

Digit	1	2	3	4	5	6	7	8	9
Percentage	0.30103	0.17609	0.12494	0.09691	0.07918	0.06695	0.05799	0.05115	0.04576

## Data Availability

All datasets are available at http://eikon.thomsonreuters.com/index.html, accessed on 29 April 2021.

## Abbreviations

The following abbreviations are used in this manuscript:

BL	Benford’s Law
BSE	Bucharest Stock Exchange
IFRS	International Financial Reporting Standards
